# Extracts from *Hericium erinaceus* relieve inflammatory bowel disease by regulating immunity and gut microbiota

**DOI:** 10.18632/oncotarget.20689

**Published:** 2017-09-06

**Authors:** Chen Diling, Yang Xin, Zheng Chaoqun, Yang Jian, Tang Xiaocui, Chen Jun, Shuai Ou, Xie Yizhen

**Affiliations:** ^1^ State Key Laboratory of Applied Microbiology Southern China, Guangdong Provincial Key Laboratory of Microbial Culture Collection and Application, Guangdong Open Laboratory of Applied Microbiology, Guangdong Institute of Microbiology, Guangzhou 510070, China; ^2^ Department of Pharmacy, The Fifth Affiliated Hospital of Guangzhou Medical University, Guangzhou 510700, China; ^3^ Departerment of Infertility and Sexual Medicine, The Third Affiliated Hospital of Sun Yat-sen University, Guangzhou 510631, China; ^4^ Guangdong Yuewei Edible Fungi Technology Co. Ltd, Guangzhou 510070, China

**Keywords:** anti-inflammatory, gut microbiota, Hericium erinaceus, immune-enhancing effect, inflammatory bowel disease

## Abstract

*Hericium erinaceus* (HE), a traditional edible mushroom, is known as a medicine food homology to ameliorate gastrointestinal diseases. To investigate whether HE is clinically effective in alleviating inflammatory bowel disease (IBD), HE extracts (polysaccharide, alcoholic extracts and whole extracts were prepared using solvent extraction methods) were administrated for 2 weeks in rats with IBD induced by trinitro-benzene-sulfonic acid (TNBS) enema (150 mg/kg). Significant clinical and histological changes in IBD rats were identified, including damage activity, common morphous and tissue damage index scores in colonic mucosa and myeloperoxidase (MPO) activity. The damage activity, common morphous and tissue damage index scores in colonic mucosa (*P* <0.05) were improved, MPO activities were decreased. Inflammatory factors were also differentially expressed in colonic mucosa in IBD rats, including serum cytokines, Foxp3 and interleukin (IL)-10 were increased while NF-κB p65 and tumor necrosis factor (TNF)-α were decreased (*P* <0.05), and T cells were activated (*P* <0.05), especially in the alcohol extracts-treated group. We also found that the structure of gut microbiota of the *H. erinaceus* extracts-treated groups changed significantly by compared with the model group. Further studies revealed that the polysaccharides in HE extracts may play a prebiotic role, whereas the alcoholic extracts show bactericidin-like and immunomodulatory effects. Taken together, we demonstrated that *H. erinaceus* extracts could promote the growth of beneficial gut bacteria and improve the host immunity *in vivo* IBD model, which shows clinical potential in relieving IBD by regulating gut microbiota and immune system.

## INTRODUCTION

Inflammatory bowel diseases (IBDs), including ulcerative colitis (UC) and Crohn’s disease, are characterized by recurring symptoms of abdominal pain, diarrhea, pus, and bleeding [1]. The incidence and prevalence rates of IBDs have been reported to be increasing worldwide [2]. The etiology and pathogenesis of IBD are still unclear; hereditary susceptibility, immune dysfunction, infection, and psychological and environmental factors are predicted to have a certain relationship [3]. Unfortunately, treatments for IBDs are very limited, including mainly 5-aminosalicylic acid preparations [4], corticosteroids, immunosuppressive agents, and some biological agents [5–7]; however, these drugs have different limitations and also have side effects more or less for the patients. Therefore, new drugs and strategies are needed to enhance the clinical responses and outcomes.

Recently, gut microbiota is considered an important factor in the progress of IBD [8–10], although the etiology and pathogenesis of IBD are still unclear and varied. In genetically susceptible individuals, the gut mucosal integrity is damaged and the microbial antigens escape through the epithelial barrier more easily, thereby activating inappropriate immune response or underlying chronic inflammation [11]. The gut microbiota plays an important role in maintaining the intestinal balance by activating the natural Toll-like receptors in the damage and repair process of intestinal mucosal integrity in patients with IBDs [12, 13]. Previous studies indicated that enteric flora disturbance could cause IBDs in mice and reduce the microbial diversity in patients, as when the intestinal bacteria increased, but some of the Bacteroidetes and Firmicutes decreased [8, 14]. How the enteric flora disturbance influences the IBDs is still unclear. It might be related to invasion of some pathogens and reduction in some protective bacteria, resulting in the activation of some abnormal immune cells, destruction of Th1- and Th17-mediated immune responses, increase in the mucous membrane permeability, loss of immune tolerance function, and so on [8, 14]. Previous studies showed that probiotic bacteria might be useful in preventing and treating acute and chronic conditions including antibiotic-associated diarrhea and IBDs [15]. Variation in host physiology caused by different diet, age, lifestyle, genetics and other factors might have a significant impact on microbiota [16–19]. At present, the outcomes of clinical and animal studies on gut microbiota lack consistency, and to understand the impacted gut microbiota function factors need an improved study design and better control over microbiota-mediated effects.

The edible and medicinal fungi are well known and widely used as part of traditional diet and herbs in Asia. In the last decades, the medicinal properties of these fungi have become the core of intense researches in naturally produced pharmaceuticals [20, 21]. *Hericium erinaceus*, belonging to the division Basidiomycota and class Agaricomycetes, is both an edible and medicinal mushroom. It is a popular delicacy across the continents and replaces pork or lamb in Chinese vegetarian cuisine. It is rich in active constituents of diterpenoid compounds, steroids, polysaccharides, and other functional ingredients [22]. It is reported that *H. erinaceus* extracts have antimicrobial activities against both antibiotic-resistant and nonresistant (susceptible) pathogenic bacteria [21, 23–26], especially *Helicobacter pylori* [27, 28], a human gastrointestinal pathogen causing adverse effects including ulcers.

IBD models are diverse [29, 30], and can be broadly categorized into four kinds: chemically induced, biologically induced, genetic (including congenic and genetically modified animals), and cell transfer models. A chemical, 2, 4, 6-trinitrobenzene sulfonic acid (TNBS), is administered rectally in the form of an enema to mice or rats [31]. It is administered in combination with ethanol, which disrupts the mucous barrier. It is generally thought that TNBS induces colitis by haptening -proteins within the gut, making them preferential targets for immune cells. In this study, a rat and mice colitis model was induced with a TNBS enema, and *H. erinaceus* extracts (polysaccharide, alcoholic extracts, and whole extracts) were administered intragastrically for 2 weeks to evaluate the treatment effect, with the aim to seek novel and effective drugs or foods from natural resources to relieve the symptoms.

## RESULTS

### Clinical improvements by *H*. *erinaceus* extracts administration

**Figure 1:**
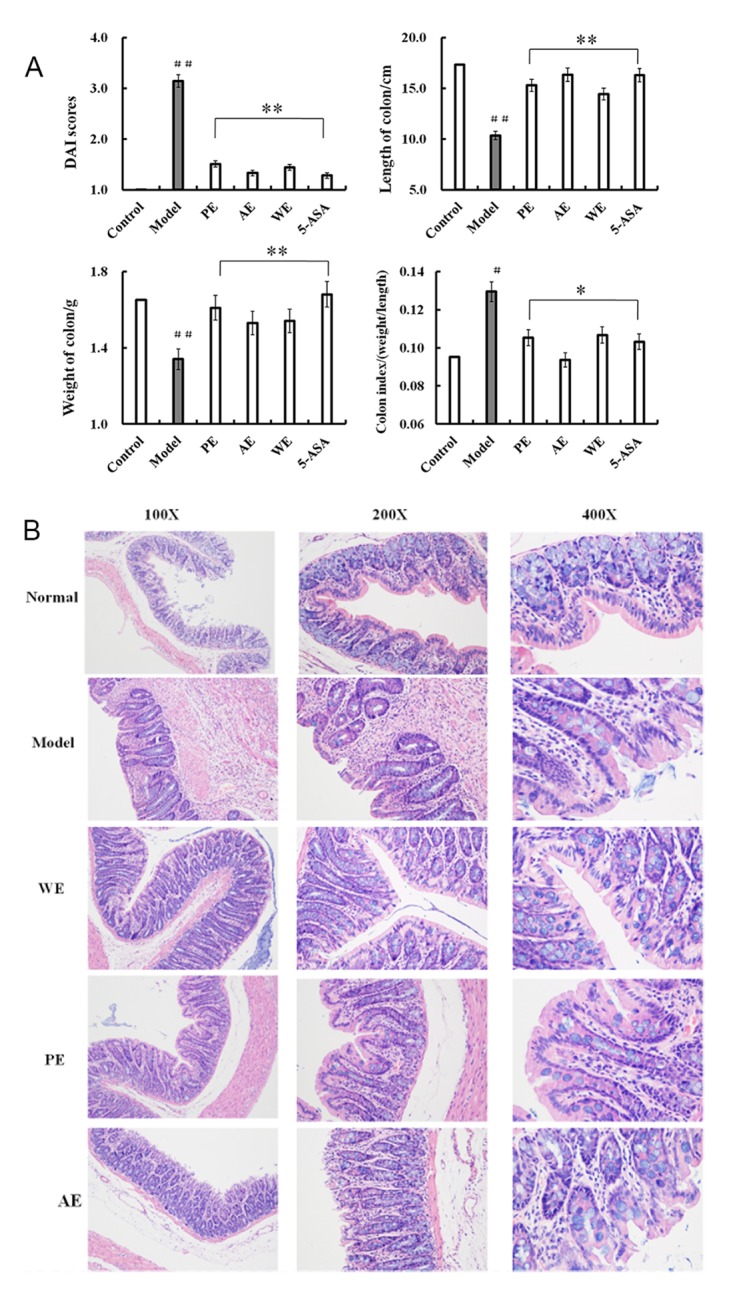
DAI scores and histopathological changes in the colon of rats in differently treated groups after induction by TNBS DAI scores were calculated according to the **(A)** weight loss, stool consistency, and blood in feces, length of the colon, weight of the colon, and the colon index (weight/length), with six rats per group; **(B)** histopathological changes in the colon. Normal, group without any treatment; model, the TNBS-induced group; WE, the whole extract-treated group after TNBS induction; PE, the polysaccharides extract-treated group after TNBS induction; AE, the alcohol extracts-treated group after TNBS induction. Values are expressed as means ± standard deviation. ^#^*P* < 0.05 vs the control group, *P < 0.05, **P < 0.01 vs the model group, indicating significant differences compared with the model group.

After treatment with TNBS enema, the rats in all the groups except for the control were anepithymia with reduced activity, lethargy, weight loss, and ruffled fur, along with bloody stools or occult blood. However, these symptoms disappeared from day 9 or day 10. The results of DAI after 14 days of treatments are shown in Figure 1A. The rats in the *H. erinaceus* extracts-treated groups showed significant improvement compared with the TNBS-treated group.

### Histological improvements by *H*. *erinaceus* extracts administration

After 14 days of treatment, the rats were killed and their colons were dissected. Hematoxylin and eosin (H&E)-stained sections showed mucosal erosions and ulceration in the TNBS-induced group (Figure 1, model group) compared with the normal group (Figure 1, normal group). The lesions were situated in the mucosa and submucosa, with architectural disruption of the colonic crypts and an inflammatory infiltrate in the mucosa including neutrophils, lymphocytes, and macrophages. Variable crypt architecture was observed, from distorted and dilated to decrease and absent. In addition, occasional crypt abscesses were considered. Injuries of the *H. erinaceus* extracts-treated groups were significantly less severe compared with the TNBS-induced group.

### Amelioration of the expression levels of serum cytokines

**Figure 2:**
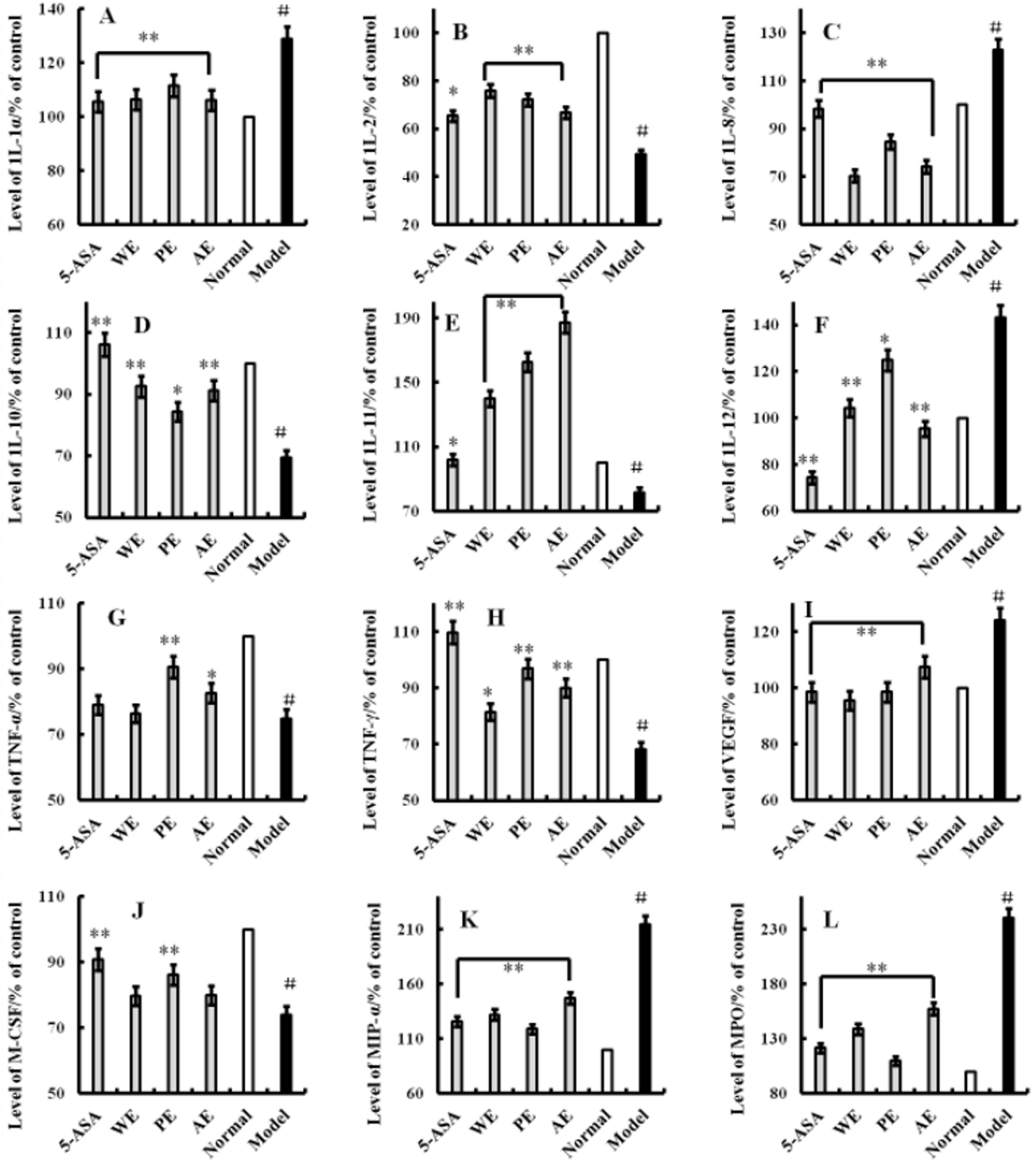
Effects of *H. erinaceus* extracts on TNBS-induced rats Rats in the normal and model groups were induced by TNBS; WE, the whole extract-treated group after TNBS induction; PE, the polysaccharides extract-treated group after TNBS induction; AE, the alcohol extract-treated group after TNBS induction. The positive control group was treated with 100 mg/(kg·day) of 5-aminosalicylic acid. After treatment for 14 days, the cytokines IL-1α **(A)**, IL-2 **(B)**, IL-8 **(C)**, IL-10 **(D)**, IL-11 **(E)**, IL-12 **(F)**, TNF-α **(G)**, TNF-γ **(H)**, VGEF **(I)**, M-CSF **(J)**, MIP-α **(K)** and MPO **(L)** were produced. The assays were carried out according to the procedures recommended in the enzyme-linked immunosorbent assay kit manual. Values were means ± standard deviation of three independent experiments. ^#^*P* < 0.05 vs the normal group, *P < 0.05, **P < 0.01 vs the TNBS-treated group, indicating significant differences compared with the LPS-treated group.

After 14 days of treatment, the serum levels of IL-1α, IL-2, IL-8, IL-10, IL-11, IL-12, TNF-γ, TNF-α, VGEF, MIP-α, and M-CSF were monitored. As shown in Figure 2, the proinflammatory cytokine levels of IL-1α (Figure 2A), IL-8 (Figure 2C), IL-12 (Figure 2F), TNF-α (Figure 2G), VGEF (Figure 2I), and MIP-α (Figure 2K) increased (*P* < 0.05) after the rats were injected with 150 mg/kg of TNBS enema, while the anti-inflammatory cytokines levels of IL-2 (Figure 2B), IL-10 (Figure 2D), IL-11 (Figure 2E), TNF-γ (Figure 2H), and M-CSF (Figure 2J) decreased, compared with the normal group (*P* < 0.05). This indicated that the inflammation was induced with the TNBS enema. After treatment with *H. erinaceus* extracts, all the cytokines levels were restored to near normal; some anti-inflammatory cytokines were secreted significantly and were better than the positive control group (*P* < 0.05). All the results demonstrated that the *H. erinaceus* extracts could be potential drugs and food resources for treating IBDs.

### Decrease in the expression of tissue myeloperoxidase

Neutrophils were the main inflammatory cells in the damaged colonic tissue, leading to an increased level of myeloperoxidase (MPO). The MPO level was decreased according with the degree of inflammation, as shown in Figure 2L. The MPO level in the TNBS-induced rats was significantly higher than that in the normal group (*P* < 0.05). The MPO level in tissues from *H. erinaceus* extracts-treated groups was significantly lower compared with the TNBS-induced group (*P* < 0.05). The result indicated that extracts from *H. erinaceus* reduced the degree of inflammation of the colon in IBD rats.

### Amelioration of the expression of colonic Foxp3, NF-κB p65, TNF-α, and IL-10 proteins

**Figure 3:**
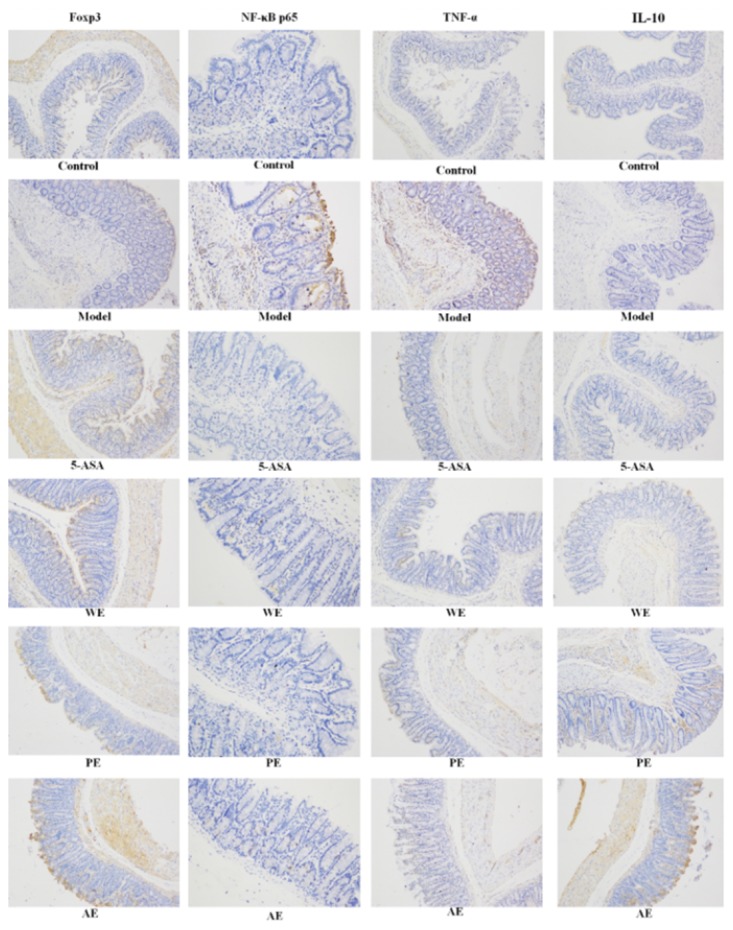
Immunohistochemistry staining of Foxp3, NF-κB p65, TNF-α, and IL-10 in the colons of different experimental groups (×200) in IBD rats Rats in the normal without nothing treatment, and model groups were induced by TNBS; WE, the whole extract-treated group after TNBS induction; PE, the polysaccharides extract-treated group after TNBS induction; AE, the alcohol extract-treated group after TNBS induction.

All sections were observed under the same conditions using a light microscope (Figure 3). The brown particles in the nucleus or in the cytoplasm were considered as positive cells. The proportion of Foxp3- and IL-10-positive cells in rats in the model group was significantly lower than that in the normal group (*P* < 0.05), while the levels of TNF-α and NF-κB p65 were significantly higher (*P* < 0.05). After treatment with *H. erinaceus* extracts, the proportion of Foxp3- and IL-10-positive cells significantly increased, especially in the alcoholic extracts (AE) (*P* < 0.05), compared with the model group. Nevertheless, the proportion of TNF-α- and NF-κB p65- positive cells was significantly reduced compared with the model group (*P* < 0.05). Cumulatively, these results suggested that *H. erinaceus* extracts had effective anti-inflammatory effects in IBD.

### The gut microbiota structure significantly changed by *H*. *erinaceus* extracts administration

**Figure 4:**
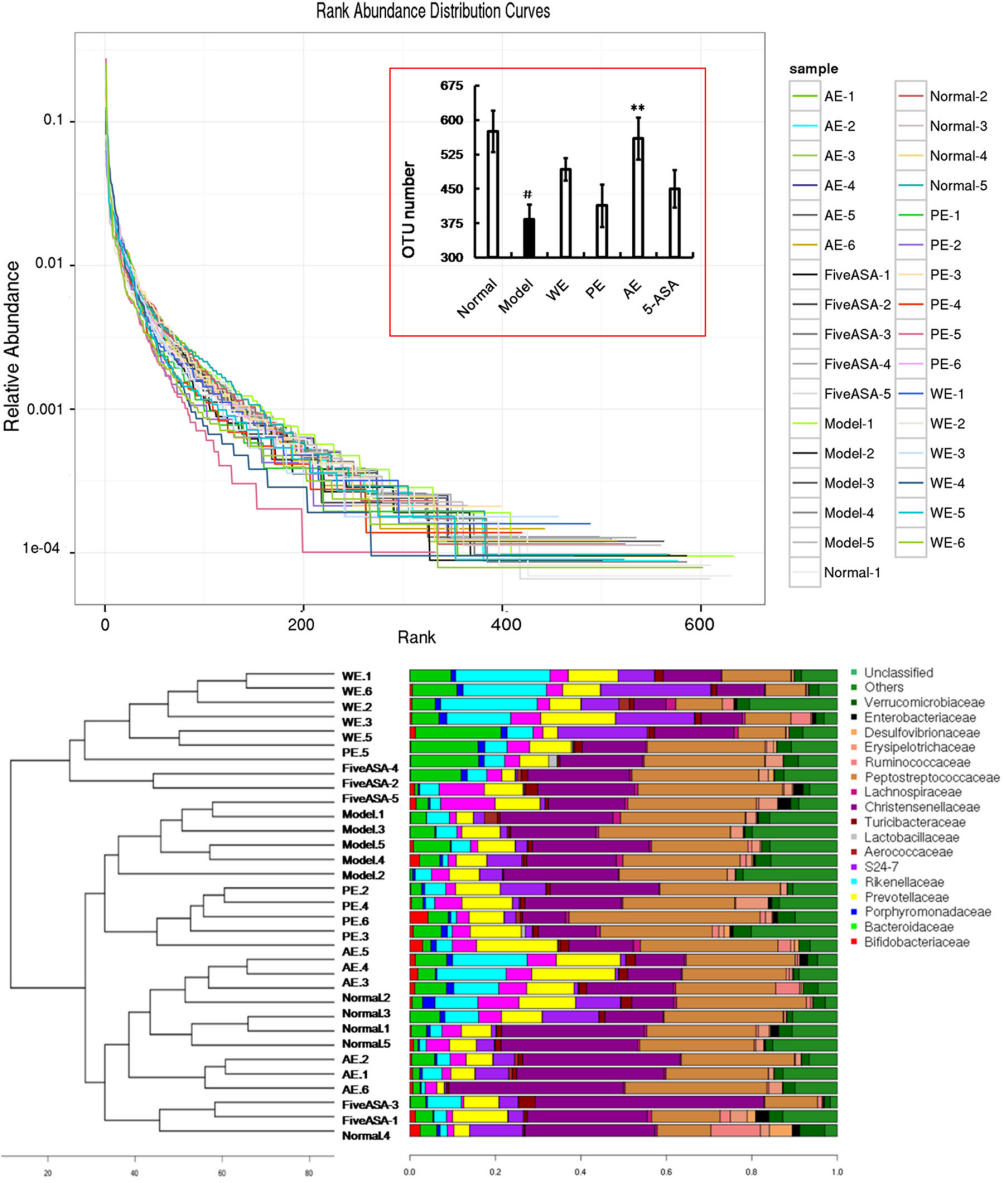
The Bray coefficient of cluster analysis and stacked figure of family composition based on the best classification level of the IBD rats induced by TNBS enema

Operational taxonomic unit (OTU) abundance and taxonomic profiles were analyzed as shown in Figure 4. We also constructed and visualized a taxonomic tree of the predominant taxa (Figure 6). All the treated groups can be clustered well using Bray-Curtis distance. The gut microbiota structure of the AE-treated group was close to the normal rats, while the PE-treated group were close to the model rats, and the WE-treated group was neither different from the normal nor the TNBS-induced group (Figure 4), which indicated that the alcoholic extracts (AE) of *H. erinaceus* may regulate the gut microbiota to the normal-like structure in the TNBS-induced IBD rats as the main active ingredients of *H. erinaceus*.

**Figure 5:**
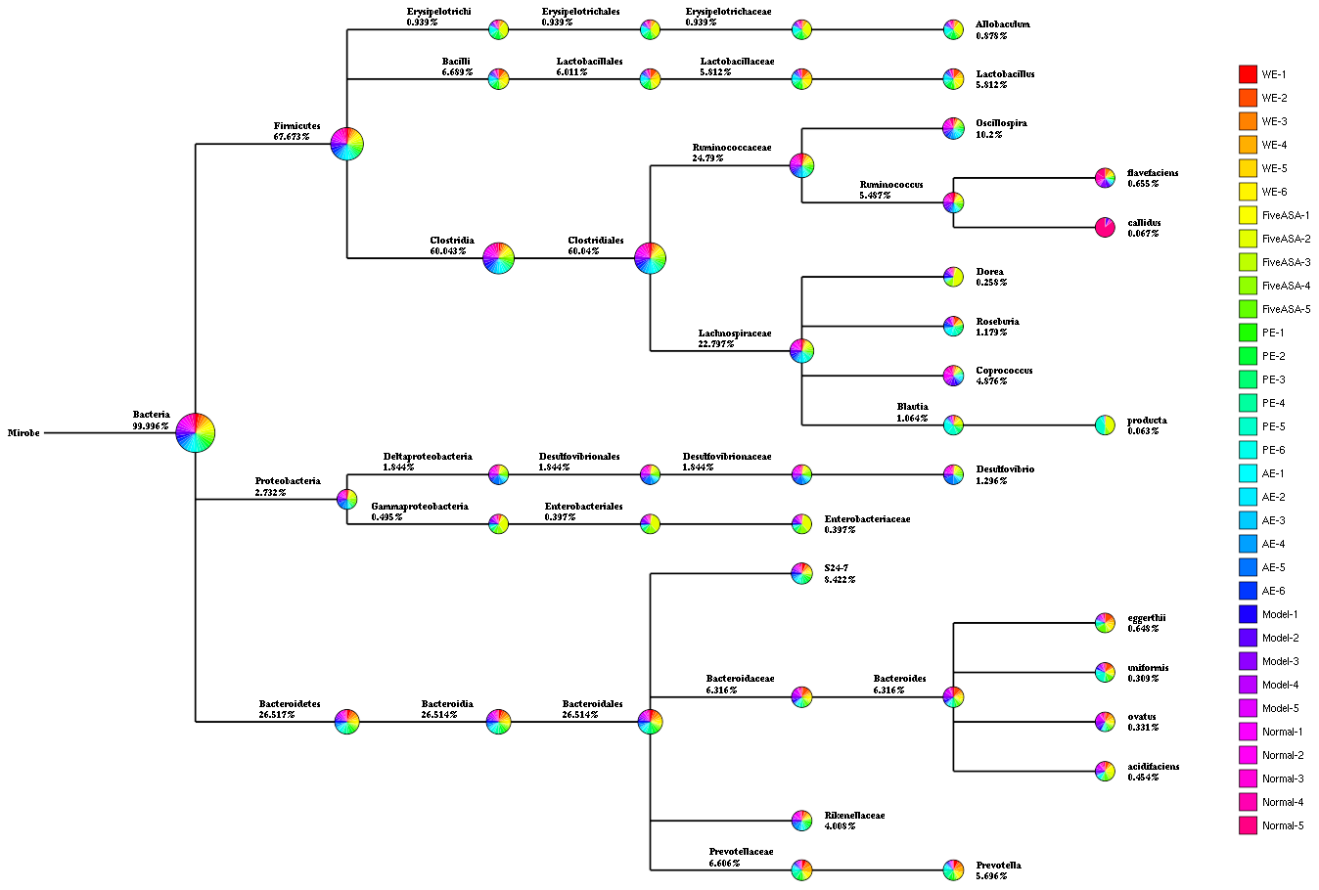
Tree species classification of the differently treated samples from IBD rats induced by TNBS enema The pie chart of different colors represents different samples in that classification unit. The size of the radius denotes the tag number accounts for the proportion of the total tag; the larger the radius, the higher the abundance. The numbers next to the pie chart represent the percentage of tag number of the percentage of the total tag.

**Figure 6:**
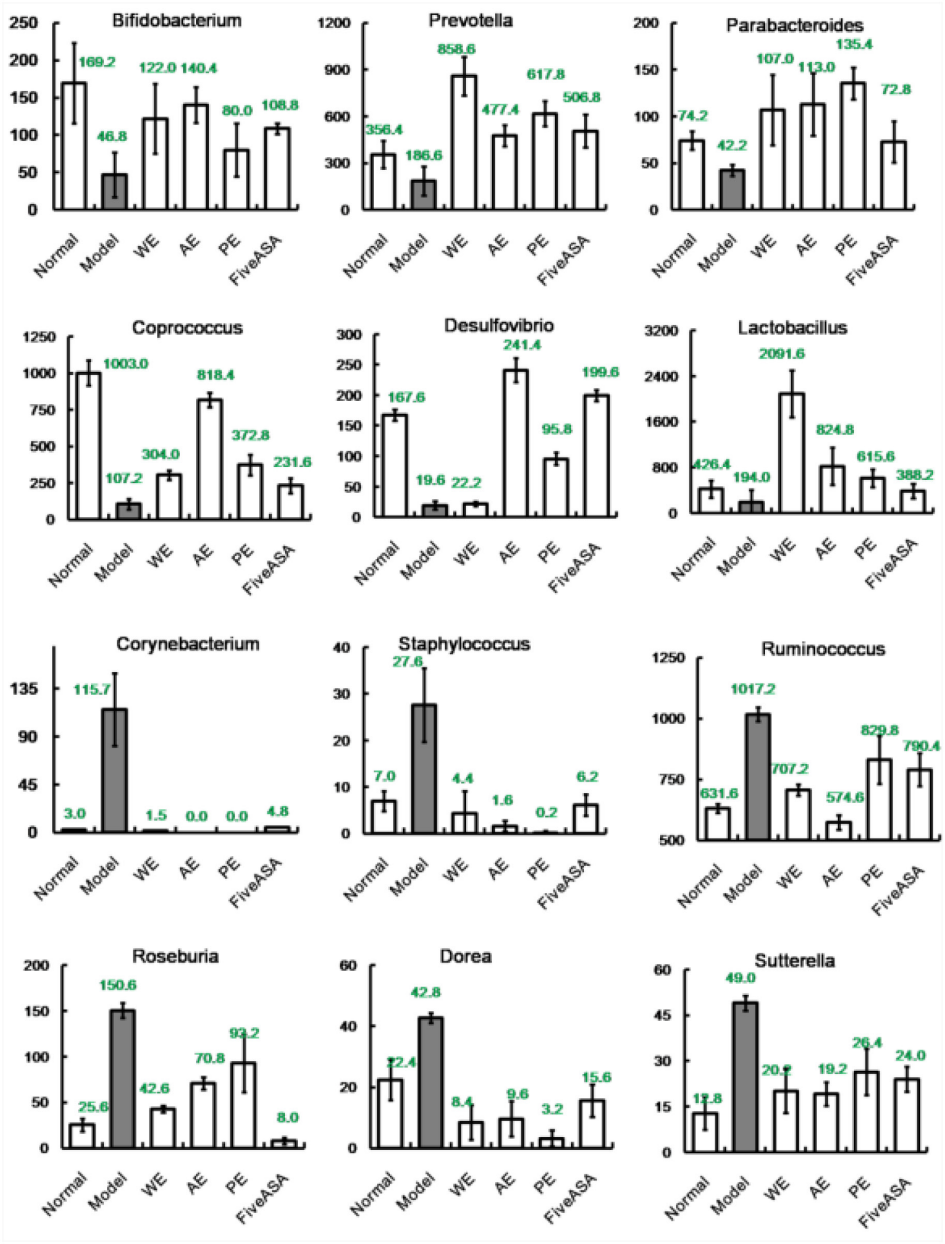
Tags with significant differences in the relative abundance of different grouping distribution of genus in IBD rats induced by TNBS enema

### Analysis of mechanical contribution to bacterial dynamic balance

Metastats analysis revealed the difference of taxonomic abundance between different groups together with FDR correction, as revealed in Figure 5. Some bacteria in the fecal samples sharply changed from the family classification level. Moreover, at the genus level, the TNBS-induced rats exhibited enrichment of potentially proinflammatory microbes, such as *Corynebacterium, Staphylococcus, Ruminococcus, Roseburia, Dorea*, and *Sutterella*, and reduction of potentially anti-inflammatory microbes, such as *Bacteroides, Bifidobacterium, Prevotella, Parabacteroides, Coprococcus, Desulfovibrio*, and *Lactobacillus* [32–34], compared with the normal group (Figure 6). However, treatment with the *H. erinaceus* extracts exhibited reduction in proinflammatory microbes and enrichment of anti-inflammatory microbes, especially in the AE-treated group. All the results indicated that the *H. erinaceus* extracts had the potential to regulate the gut microbiota structure.

### Prebiotic effect of polysaccharides in *H*. *erinaceus* extracts on TNBS-induced mice

**Figure 7:**
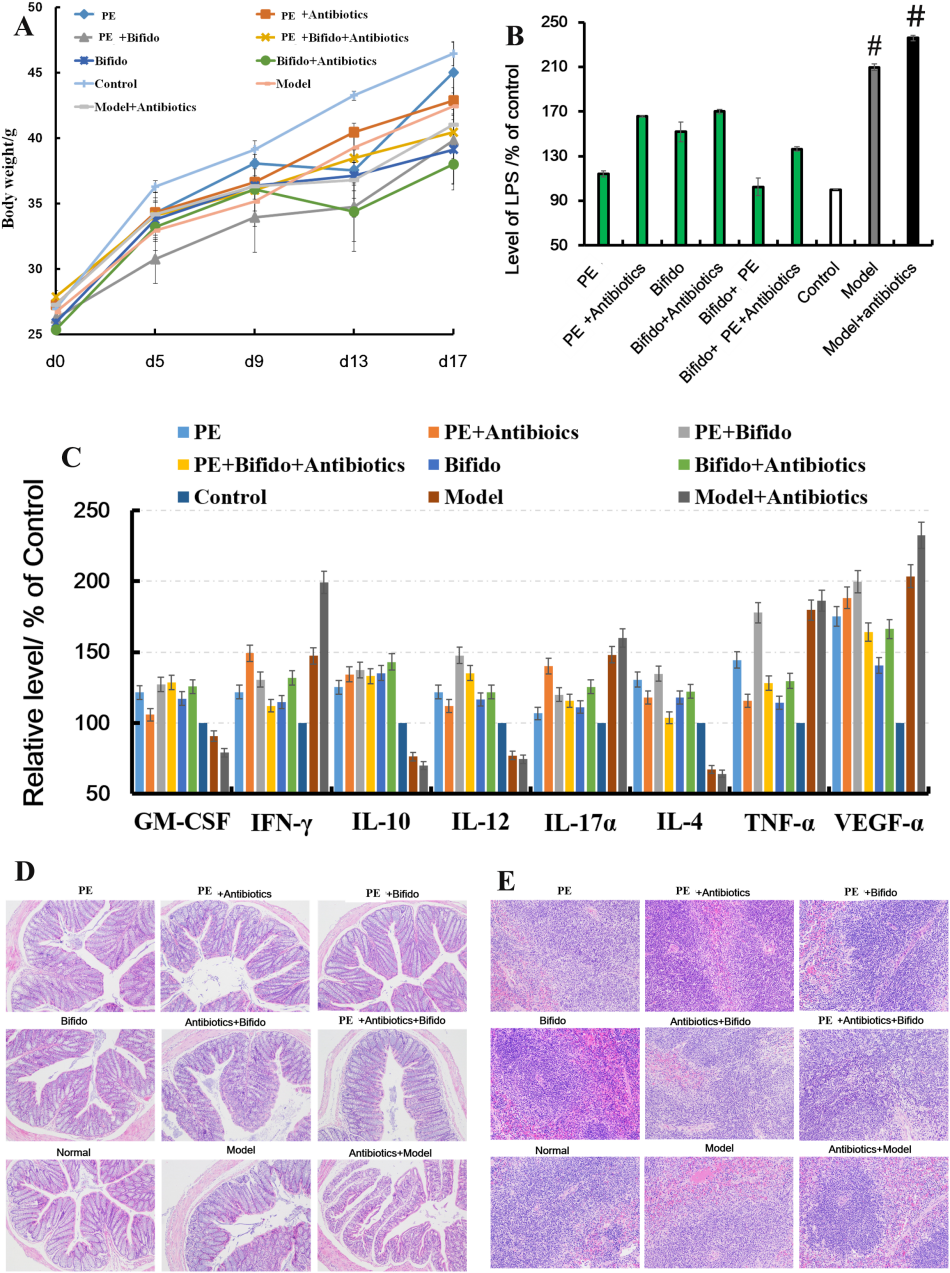
Polysaccharides extracted from *H. erinaceus* improved the pathological parameters of the TNBS-induced mice **(A)** Body weight changes; **(B)** levels of LPS in serum; **(C)** levels of cytokines GM-CSF, TNF-γ, IL-10, IL-12, IL-17α, IL-4, TNF-α, and VGEF-α in serum; **(D)** histopathological changes in the colon; and **(E)** histopathological changes in the spleen. Control is the normal without any treatments; model (Mice were induced by TNB), model and high-dose antibiotics, polysaccharides [100 mg/(kg · d)], *Bifidobacterium*, polysaccharides plus high-dose antibiotics, polysaccharides plus *Bifidobacterium*, *Bifidobacterium* plus high-dose antibiotics, and polysaccharides plus *Bifidobacterium* plus high-dose antibiotics.

**Figure 8:**
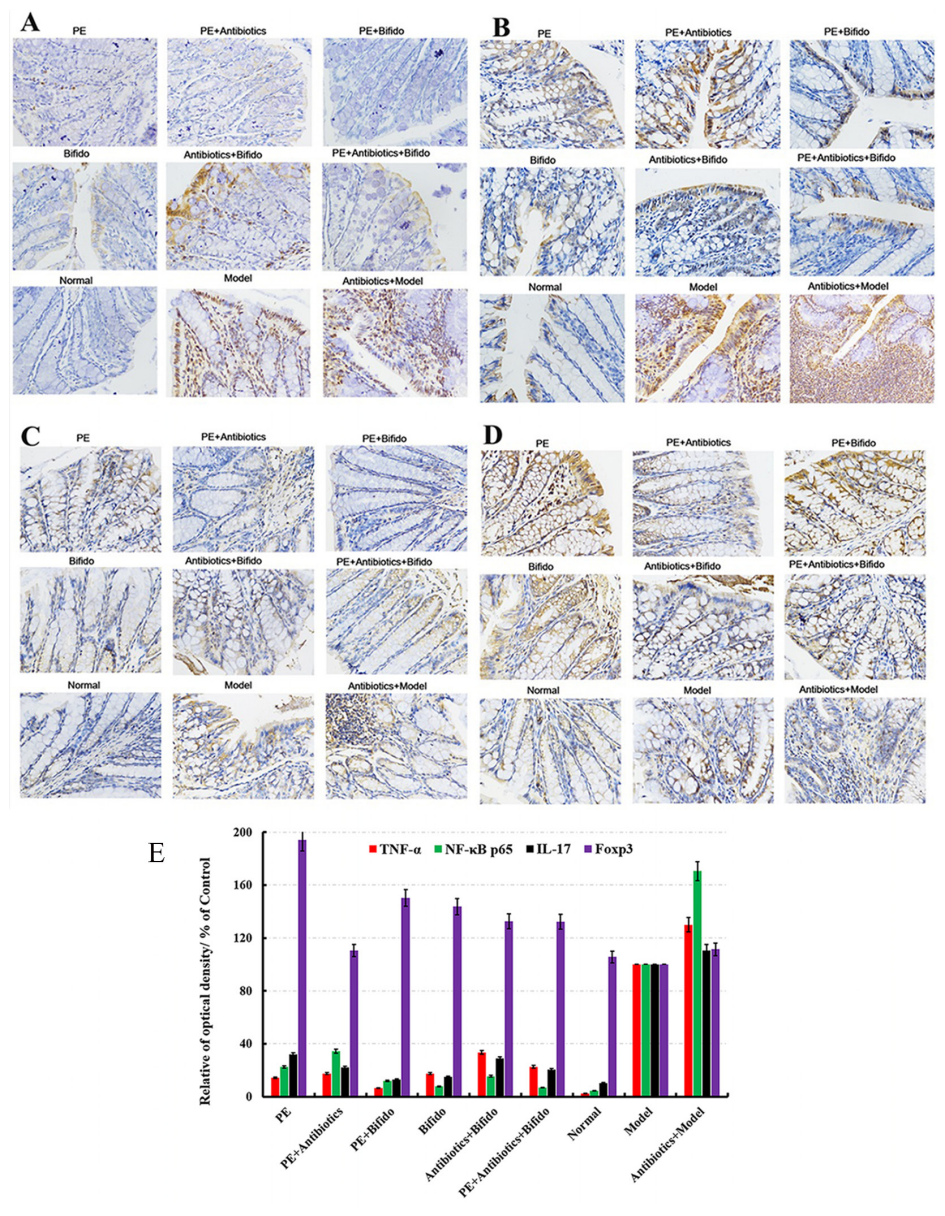
Immunohistochemistry staining of **(A)** TNF-α, **(B)** NF-κB p65, **(C)** IL-17, **(D)** Foxp3 and the mean optical density values were used for analyzing the results of immunohistochemistry **(E)** in the colons of different experimental groups in IBD mice induced by TNBS enema after treatment with polysaccharides extracted from H. erinaceus.

An IBD mice model was prepared after treatment with broad-spectrum antibiotic for 1 week to extensively evaluate the prevention and cure effects of polysaccharides on IBD. As shown in Figure 7, the colonic tissues were seriously damaged in the TNBS-combined antibiotics-treated groups, and some cytokines such as GM-CSF, TNF-γ, IL-10, IL-12, IL-17α, IL-4, TNF-α, VGEF-α, and LPS were far from the control, indicating that without the microbiota the intestinal mucosa (Figure 7D) could be easily destroyed (Figure 7C). When treated with *H. erinaceus* polysaccharides, especially the combined *Bifidobacterium-*treated groups, all the cytokine levels were restored to near normal, and even some clinical parameters were significantly better than those in the control group (*P* < 0.05). Moreover, the immunohistochemical staining showed that the expression of NF-κB, TNF-α, and IL-17 in the polysaccharide- and *Bifidobacterium-*treated groups decreased compared with the model (*P* < 0.05) and TNBS-combined antibiotics-treated groups (*P*<0.01); however, the expression of Foxp3 increased (*P* < 0.01), as shown in Figure 8.

**Figure 9:**
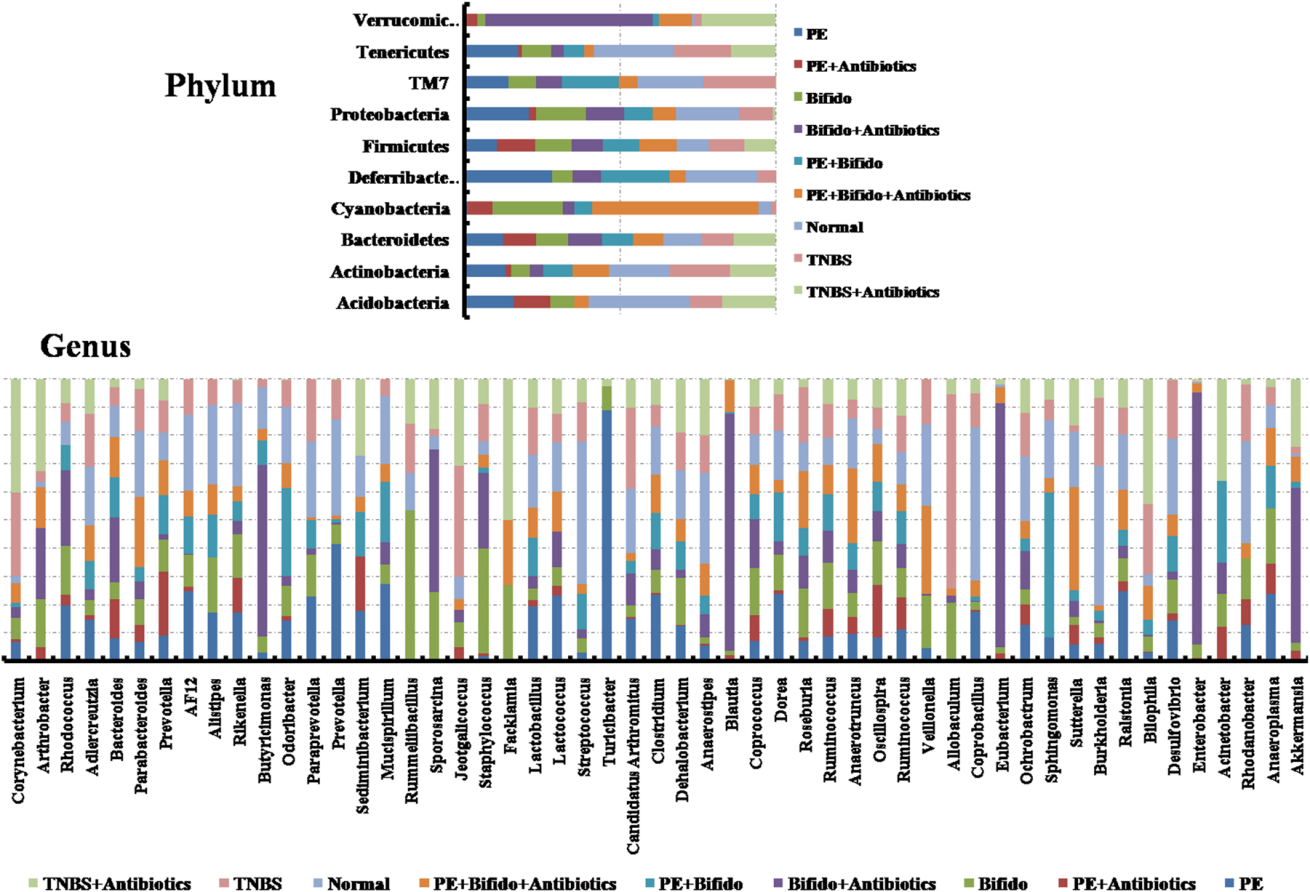
Polysaccharides extracted from *H*. *erinaceus* showed a good prebiotic effect on TNBS-induced mice.

Further statistical analysis revealed that the polysaccharides could significantly enhance the settling down of *Bifidobacterium* (*P* < 0.05; Figure 9). Moreover, the other prebiotics increased. The results showed that the *H. erinaceus* polysaccharides could be useful as prebiotics.

## DISCUSSION

Mushrooms are generally a good source of nutrients and health-promoting compounds. The findings of the present study showed that the *H. erinaceus* extracts (polysaccharide, alcoholic extracts, and whole extracts) significantly improved the structure of gut microbiota. Some symptoms of IBD such as common morphous and tissue damage index scores in colonic mucosa improved (*P* < 0.05); the MPO activity decreased; the levels of serum cytokines, including IL-1α, IL-2, IL-8, IL-10, IL-11, IL-12, TNF-γ, TNF-α, VGEF, MIP-α, and M-CSF levels, improved to near normal; and the expression of Foxp3, NF-κB p65, TNF-α, and IL-10 in colonic mucosa improved (*P* < 0.05). These results clearly indicated that the *H. erinaceus* extracts could promote the growth of probiotic bacteria, improve immunity, and give positive results in the experimental models of IBD.

**Figure 10:**
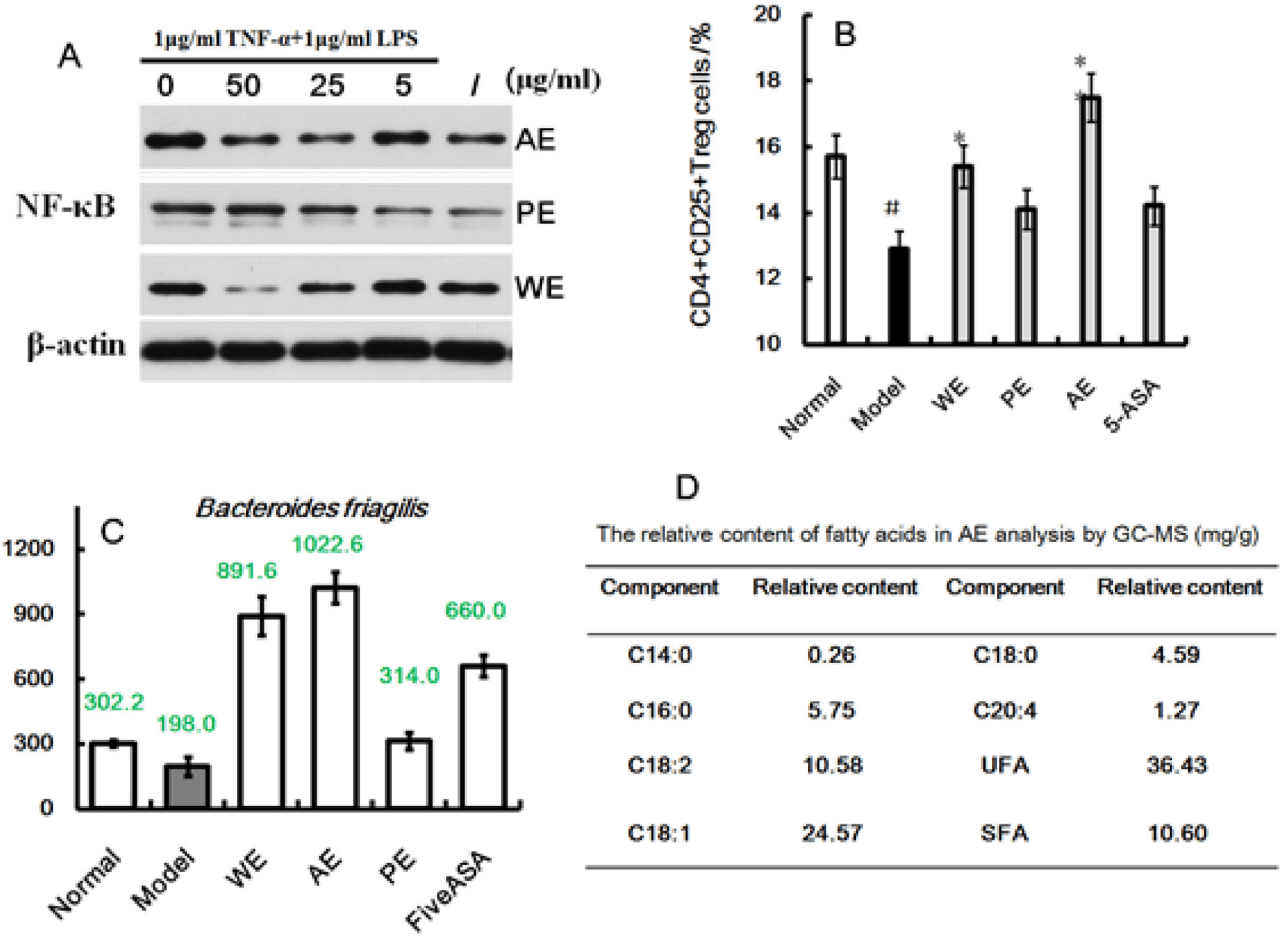
Effect of AE on the TNF-α- and LPS-induced Caco-2 cells **(A)**, CD^4+^CD^25+^Treg cells in TNBS-induced IBD rats induced by TNBS enema **(B)**, relative abundance of *B. friagilis* differently treated IBD rats **(C)**, and the relative content of fatty acids in AE from *H. erinaceus* analysis by GC–MS **(D)**.

Immune factors play a predominant role in the pathogenesis of IBDs. Cytokines, including IL-1, IL-2, IL-12, TNF-α, VGEF, and MIP-α, were proinflammatory, while IL-8, IL-10, IL-11, TNF-γ, and M-CSF were anti-inflammatory. These cytokines have various biological activities such as transferring molecules, mainly regulating the immune response, participating in the immune cell differentiation development and tissue repair, interfacing inflammation, and stimulating hematopoietic function and other functions. Anti-inflammatory and immunosuppressive treatments are well known for reducing and limiting the damage caused by IBD. Studies have shown that the inflammatory mediators and the pathological changes in colonic mucosa are mainly affected in IBD. The interleukins, such as IL-1, IL-4, IL-6, IL-8, and IL-10, were considered to be closely associated with UC. TNF-α helps in the infiltration of the inflammatory cells into the bowel, increasing the intestinal mucosal inflammation; promoting the release of platelet-activating factor, leukotriene, and oxygen free radical; and inducing nitric oxide synthesis leading to cell damage [35]. TNF-α, with its accomplice IL-6, induces the thrombin or even microthrombus formation on intestinal mucosa [36], leading to microcirculation disorder, grievous ischemia, and anoxia. The underlying mechanism is that free radicals and TNF-α lead to the degradation of I-κB, an inhibitory protein of NF-κB, in turn resulting in NF-κB activation and induction of downstream target genes, as shown in Figure 10A. The expression of NF-κB increased after confluent cultures with 1 μg/mL TNF-α and 1 μg/mL LPS. When treated with AE, the expression levels decreased slightly, indicating that AE could down regulate the expression of NF-κB to suppress inflammation directly. In this study, the TNF-α levels were higher in the peripheral blood and colons of the TNBS-induced rats than in the normal group; also the expression of NF-κB in the colons was activated, and the levels of IL-1α, IL-2, IL-8, IL-10 were changed. However, after treatment with the *H. erinaceus* extracts, all the inflammatory responses were ameliorated.

Foxp3 is a member of the forkhead family of transcription factors critically involved in the development and function of CD^25+^ regulatory T cells (Treg). It regulates not only the expression of CD^4+^CD^25+^Treg (as a specific marker) but also the expression of CD8^+^CD28^-^Treg. It was found that the number of CD^4+^CD^25+^Treg cells decreased in the Foxp3 gene knockout or mutation mice, greatly increasing the incidence of autoimmune diseases [37]. The homologous Foxp3 mutations were proved to cause genetic disorders in humans, such as immune disorders, bowel diseases, and endocrine diseases [38]. The expression of Foxp3 was markedly inhibited in the TNBS-treated rats in the present study (Figure 3). The number of CD^4+^CD^25+^Treg cells increased in the peripheral blood of rats after treatment with the *H. erinaceus* extracts compared with the TNBS-treated group (Figure 10B, *P* < 0.05), indicating that the *H. erinaceus* extracts could activate the immune system of IBD.

A new study reported that *Bacteroides friagilis* delivered immunomodulatory molecules to immune cells via secretion of outer membrane vesicles to Treg to suppress the mucosal inflammation in IBD [39], as shown in Figure 10C. The relative abundance of *B. friagilis* was higher in the AE-treated group than in the model group (*P* < 0.05). A previous study also indicated that short-chain fatty acids led to increased Treg cell differentiation via the small intestine, and middle- and long-chain fatty acids supported Th1 and Th17 cell differentiation [40]. Abundant fatty acids were found in AE using the gas chromatography–mass spectrometry (GC-MS) analysis (Figure 10D). Thus, this study confirmed that the fatty acids in AE were one of the pharmacodynamic ingredients of IBD. Previous studies also reported that *H. erinaceus* was rich in erinacines (3-hydroxyhericenone F, hericenone G, hericenone F, and hericerin, and so on) that have antimicrobial and wound-healing properties among other therapeutic potentials [24, 26, 41, 42], which was consistent with the results of the present study. However, the details of the pharmacodynamic ingredients still need more study.

Previous studies also reported that *H. erinaceus* was rich in some physiologically important components, especially β-glucan polysaccharides. The GC-MS analysis in this study revealed that the monosaccharide composition was mannose (∼55.70%), arabinose (∼0.7%), rhamnose (∼3.75%), xylose (∼1.60%), galactose (∼5.12%), and glucose (∼33.10%). The polysaccharides were demonstrated to have a good prebiotic effect in this study, synergistically relieving inflammation and enhancing immunity (Figure 10). The expression levels of TNF-α (Figure 8A), NF-κB (Figure 8B), and IL-17 (Figure 8C) in polysaccharides plus *Bifidobacterium*-treated group were lower while the levels of Foxp3 (Figure 8D) significantly increased (*P* < 0.05) compared with the other treated groups, even more perfect than the normal, indicating that polysaccharides could activate the immune system and inhibit the reaction of inflammation; the mechanism was related to the Foxp3 T cells.

The physical health depends much on the intestinal health. Probiotics play many important roles in maintaining the intestinal health, such as making intestinal mucous membrane surface, forming biological barrier, constituting intestinal engraftment resistance to prevent bacterial pathogens, invading conditionally pathogenic bacteria, avoiding the occurrence of diseases and safeguarding the intestinal normal metabolism [43–47]. *Bifidobacteria*, a commensal microorganism found in the gastrointestinal tract, has been proved as the core of the intestinal flora, contributing a lot to human health [48, 49]. Long-term studies have shown that the abundance of *bifidobacteria* is higher in individuals who are healthy and have greater longevity than the ordinary, and they exist throughout the lifetime [50]. Several strains have been attributed beneficial traits at local and systemic levels, through pathogen exclusion or immune modulation, among other benefits [51]. This study found that the polysaccharides could significantly increase the abundance of *Bifidobacterium* (Figure 9) in the cecum contents and reduce the levels of LPS, which were mainly secreted by *Bacteroides* spp., *B. vulgatus*, and *Desulfovibrio* spp., in serum and inflammation (Figure 7B and 7C).

**Figure 11:**
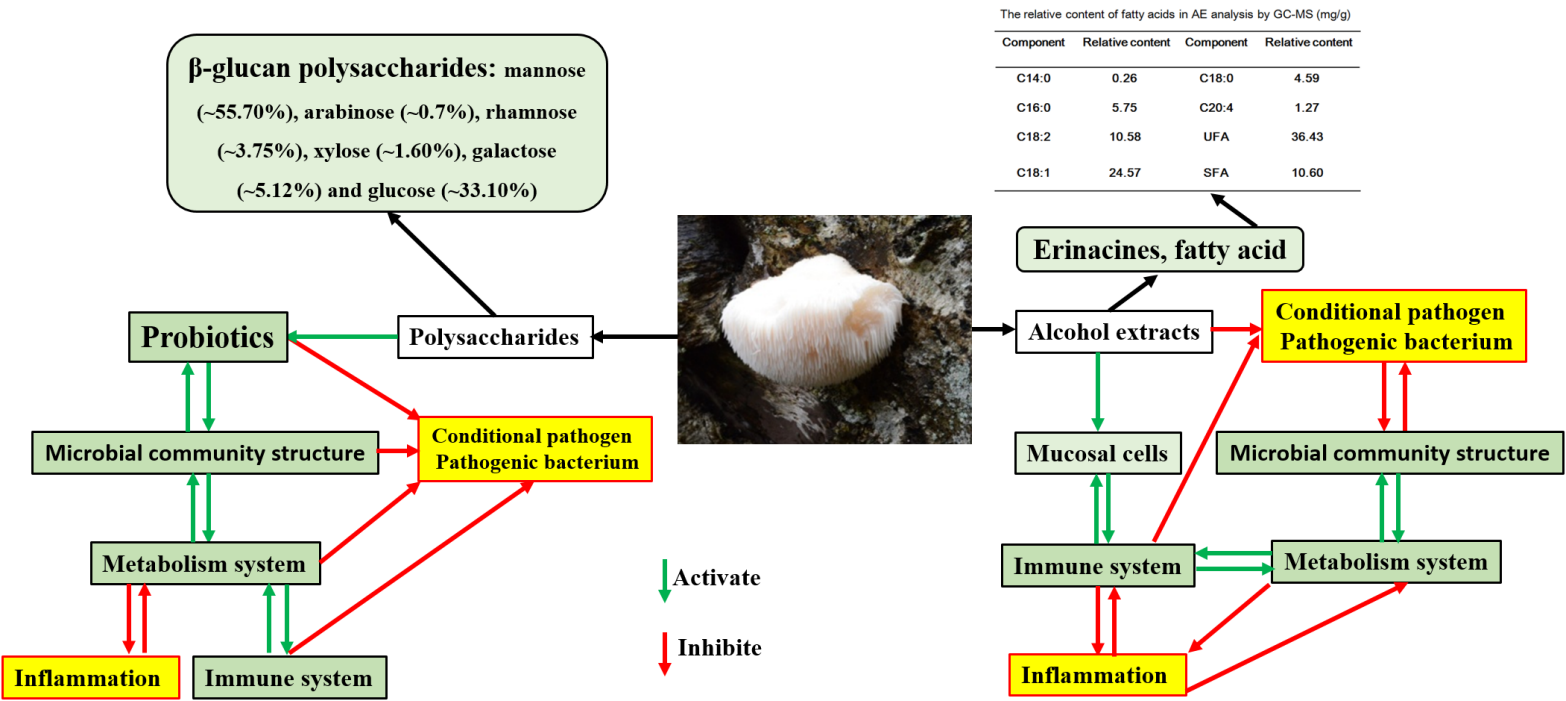
Active ingredients and mode of action of *H*. *erinaceus* for IBD prevention and treatment.

The importance of human gut microbiome in health and disease has been recognized for decades. Increasing evidence indicated that altered intestinal microbial composition and function resulted in an increased risk of IBD [52]. Previous studies showed that diet resulted in significant changes in the composition of the cecal and fecal microbiota, and found diet-dependent shifts among the dominant bacterial phyla in the intestine of mice [18, 53–55]. In humans, diet also modulated the intestinal microbiota [56–59]. Findings from many laboratories have shown that the composition and products of the gut microbiota have unexpected effects on immune and inflammatory responses [17, 60, 61], and the genes that are also believed to be involved in IBD are usually involved in immune responses, transport, or bacterial recognition [62]. By changing gut microbiota, cancer incidence can be also change, IBD is the important induced factor for malignant gastrointestinal tumor [63, 64]. In this study, the TNBS could significantly reduce the OTUs of the gut microbiota in rats (*P* < 0.05 vs the normal group), as shown in Figure 5, while treatment with *H. erinaceus* extracts recovered the number of OTUs to near normal, especially in the AE-treated groups (*P* < 0.05 vs the TNBS-induced group, *P* > 0.05 vs the normal group) and ameliorated the clinical parameters of the IBD rats. All these results indicated that *H. erinaceus* had a potential to naturally improve the structure of gut microbiota/ immunomodulatory property and could be used as a drug or functional food ingredient for IBD or other gastrointestinal bacterial infections. The active ingredients and mode of action of *H. erinaceus* for IBD prevention and treatment are shown in Figure 11. The AE containing fatty acids and erinacines (3-hydroxyhericenone F, hericenone G, hericenone F, and hericerin, ect) have antimicrobial and wound-healing properties, which can directly inhibit pathogen and repair the wound. The polysaccharides maninly influence the structure of the gut microbiota as polysaccharides for essential nutrients, and the small molecular organic acids produced from gut microbiota have many biological activities [65] but still need much more studies.

## MATERIALS AND METHODS

### Preparation of the *H. erinaceus* extracts

The whole fruiting bodies of *H. erinaceus* (commoditized strain) were collected from the Research Laboratory of Edible Mushrooms of Guangdong Institute of Microbiology, China, in May 2015, and identified by Prof. Xie Yizhen of Guangdong Institute of Microbiology using shape features and ITS sequences. They were dried at 60°C, ground into a fine powder, and averaged into two equal parts.

Ten parts of alcohol were added to one half of the aforementioned powdered substance and then refluxed for 1 h twice. The filtrate was merged and concentrated to dry at a low pressure using rotary evaporators. The residue collected was the AE, which was stored at 4°C and prepared as oral suspension with distilled water for further use. Then, eight parts of distilled water was added to the alcohol-extracted residue, soaked for 2 h, and refluxed for 1 h twice. The filtrate was merged and concentrated to 1 g/mL (equivalent to the crude drugs) at a low pressure using rotary evaporators. When the solution cooled to 45°C, the pH value was adjusted to 6.0 using diluted hydrochloric acid. The solution was then allowed to traverse through the ion exchange resin (D-900; Hebei Cangzhou Baoen Adsorption Materials Technology Co., Ltd, Hebei, China) at a speed of 1.0 Bv/h two times. The eluent was then concentrated to a low volume, cooled to room temperature, added to alcohol to a concentration of 80%, and refrigerated at 4°C for 48 h. The precipitation collected was the PE, which was stored at 4°C and prepared as oral suspension with distilled water for further use.

Ten parts of 50% aqueous alcohol (v/v) was added to the other half of the powdered substance and refluxed for 1 h twice. The filtrate was merged and concentrated to dry at a low pressure using rotary evaporators. The residue collected was the WE, which was re-suspended in distilled water and stored at 4°C for further use.

### Animals and treatments

Thirty adult male Sprague–Dawley rats (weight 180–220 g) and ninety male Kunming mice (18–22 g) were purchased from the animal center of the Guangdong Medical Laboratory Animal Center, Guangzhou, China. The animals were kept in the specific-pathogen-free laboratory of Guangdong Laboratory Animals Monitoring Institute. The rats were kept in cages with three rats per cage (30 × 30 × 20 cm^3^) in a temperature (22°C ± 1°C) and humidity (55% ± 10%) controlled room on a 12-h light/dark cycle (lights were switched off at 17:00 h). The experimental protocols were approved by the laboratory of Guangdong Laboratory Animals Monitoring Institute, and all experimental procedures conformed to the National Institutes of Health Guide for the Care and Use of Laboratory Animals. All efforts were made to minimize the number of animals used.

#### Effects on TNBS-induced IBD rats

After 7 days of adaptation period, the animals were randomly divided into five groups (six rats in each group, and three per cage): 100 mg/(kg · day), PE group; 100 mg/(kg · day), AE group; 100 mg/(kg · day, WE group; model group; and normal group, and more details showed in Table 1. The rats were fed a standard diet with free access to water. After 24 h of fasting, the rats were anesthesized using an intraperitoneal injection of 2% sodium pentobarbital (0.2 mL/100 g). They were intubated (using latex tubing of 2 mm diameter, lubricated with edible oil) through the anus, and gently pushed into the lumen to about 8.0 cm. Then, 150 mg/kg of TNBS (Sigma-Aldrich, MO, USA; dissolved in 50% ethanol) solution was injected through the latex tubing. The rats were hung upside-down for 30 s to ensure that the mixture fully seeped into the lumen without leakage. The *H. erinaceus* extracts were administered intragastrically to the TNBS-induced animals after 1 day and last 2 weeks.

**Table 1:** Experimental design of extracts effects in TNBS-induced IBD rats

Groups	Rats No.	Trinitro-benzene-sulfonic acid (TNBS) (mg/kg)	Dose (mg/kg/d)
Normal	5	/	Distilled water
Model	5	150	Distilled water
Amino saliciylic acid (5-ASA)	5	150	100
Whole extracts (WE)	5	150	100
Alcoholic extracts (AE)	5	150	100
Polysaccharide (PE)	5	150	100

#### Effects on TNBS-induced IBD mice

All mice were randomly divided into nine groups (*n* = 9): control, model, model and high-dose antibiotics, polysaccharides [100 mg/(kg · d)], *Bifidobacterium*, polysaccharides plus high-dose antibiotics, polysaccharides plus *Bifidobacterium*, *Bifidobacterium* plus high-dose antibiotics, and polysaccharides plus *Bifidobacterium* plus high-dose antibiotics. All the antibiotics (ampicillin, 2 g/L; vancomycin, 1.0 g/L; neomycin, 2 g/L; and metronidazole, 2 g/L) were given for 7 days, then IBD was induced with a TNBS (150 mg/kg) enema, and more details showed in Table 2. After 7 days of drug treatment, the mice were induced again with TNBS enema, followed by another 4 days of drug treatment.

**Table 2:** Experimental design of prebiotic effect of polysaccharides from *H. erinaceus* extracts in TNBS-induced mice

Groups	Mice No.	TNBS (mg/kg)	Dose
Control	9	/	Distilled water
Model	9	150	Distilled water
Model & high-dose antibiotics	9	150	0.2 ml
Polysaccharides	9	150	100 mg/kg/d
*Bifidobacterium*	9	150	10^9^ CFU
Polysaccharides & high-dose antibiotics	9	150	100 mg/kg/d
Polysaccharides & *Bifidobacterium*	9	150	100 mg/kg/d+10^9^ CFU
*Bifidobacterium* & high-dose antibiotics	9	150	10^9^ CFU +0.2 ml
Polysaccharides & *Bifidobacterium* & high-dose antibiotics	9	150	100 mg/kg/d+10^9^ CFU +0.2 ml

Notes: Antibiotics (ampicillin, 2 g/L; vancomycin, 1.0 g/L; neomycin, 2 g/L; and metronidazole, 2 g/L); *Bifidobacterium.* was 10^9^ CFU.

After treatment, the mice were anesthesized by an intraperitoneal injection of 2% sodium pentobarbital (0.25 mL/100 g), and the blood plasma was collected by the abdominal aortic method and the serum by centrifugation (1500 rpm, 10 min). Then, the serum was subjected to analysis to detect the production of cytokines GM-CSF, TNF-γ, IL-10, IL-12, IL-17α, IL-4, TNF-α, and VGEF, and the levels of LPS. The colons and spleens obtained from the rats were fixed in 4% paraformaldehyde at pH 7.4 for further pathological observation, and the cecum contents were collected for 16s rRNA analysis.

### General status observations and colon visceral organ index assay

The percentage weight loss, characteristics of the stool, and the presence of blood in the stool were recorded to derive a Disease Activity Index (DAI). The scoring criteria were performed by three persons, all blinded to the experimental groups before DAI scoring, as described in a previous study [66].

The weight of the colon of each rat or mouse was recorded, and then the colons were stored in 4% paraformaldehyde or frozen as soon as possible for further analysis. The colon index (the weight of the colon/the length of the colon) was calculated.

### Histopathological examinations

Histological changes were evaluated by H&E analysis and immunohistochemically stained sections. The colon samples were processed according to the protocols described in a previous study [67], as well as for immunohistochemical analysis of Foxp3, TNF-α, IL-10, and NF-κB p65 (1:100).

### Gut microbiota analysis

#### Bacterial DNA isolation

Fresh fecal samples were collected before the fasting of the rats and stored at -80°C. The total bacterial DNA was extracted from 0.25 g of the fecal sample using the repeated bead beating method described by Favier with modifications as described in a previous study [65].

### 16S rRNA gene sequencing

Fresh fecal samples were collected before the fasting of the rats and stored at -80°C. Frozen microbial DNA isolated from mice cecal sample with total masses ranging from 1.2 to 20.0 ng were stored at -20°C. The microbial communities of V3 and V4 region 16S rRNA genes were amplified using the forward primer 5’-ACTCCTACGGGAGGCAGCA-3’ and the reverse primer 5’-GGACTACHVGGGTWTCTAAT-3’. Each amplified product was concentrated via solid-phase reversible immobilization and quantified by electrophoresis using an Agilent 2100 Bioanalyzer (Agilent). After quantification of DNA concentration using Nanodrop, each sample was diluted to a concentration of 1 × 10^9^ mol/μL in the Tris-EDTA buffer and pooled. Then, 20 μL of the pooled mixture was used for sequencing with the Illumina MiSeq sequencing system according to the manufacturer's instructions. Raw pyrosequencing reads obtained from the sequencer were denoised using the Titanium PyroNoise software. The resulting pyrosequencing reads were analyzed by common analysis methods [67].

#### Microbiota classification

Raw pyrosequencing reads obtained from the sequencer were denoised using the Titanium PyroNoise software. The resulting pyrosequencing reads were analyzed as described in a previous study [67].

### Enzyme-linked immunosorbent assay

After 14 days of treatment, the rats or mice were anesthetized using an intraperitoneal injection of 2% sodium pentobarbital (0.25 mL/100 g), decapitated, and dissected. The blood plasma was collected by the abdominal aortic method and the serum by centrifugation (1500 rpm, 10 min). Then, the serum was subjected to analysis for the production of cytokines interleukin (1L)-1α, 1L-2, 1L-8, 1L-10, 1L-11, and IL-12; tumor necrosis factor (TNF)-γ and TNF-α; vascular endothelial growth factor (VGEF); human macrophage inflammatory protein-1α (MIP-α); and macrophage colony-stimulating factor (M-CSF) and so on. All the assays were carried out according to the procedures recommended in the enzyme-linked immunosorbent assay kit (Shanghai Bogoo Biological Technology Co., Ltd, China) manual.

### Determination of the MPO activity

The myeloperoxidase (MPO) colorimetric kit (Jiancheng Bioengineering Institute, China) was used to measure the MPO activity in colon tissue homogenates to evaluate the severity of UC using the thiobarbituric acid method.

### Caco-2 cell culture

Caco-2 cells (a human colonic cell line; ATCC, MD, USA) were cultured in the Dulbecco’s modified Eagle medium supplemented with 12% fetal bovine serum (FBS) and passaged twice weekly. For analysis, 10^6^ cells were plated in 24-well plates in a medium containing 12% FBS at 37°C in an atmosphere of 5% CO_2_ and relative humidity of 90% and allowed to rest for 24 h. Confluent cultures were stimulated with 1 μg/mL TNF-α and 1 μg/mL LPS added to the medium. The cells were harvested after 24 h.

### Statistical analysis

All data were expressed as means plus standard deviations of at least three independent experiments. The significant differences between treatments were analyzed by one-way analysis of variance followed by Dunnett’s test in order to detect inter-group differences. A difference was considered statistically significant if the *p* value was less than 0.05. Statistical Package was used the SPSS (Abacus Concepts, CA, USA) and Prism 5 (GraphPad, CA, USA) software.

## Abbreviations

HE: *Hericium erinaceus*
IBD: inflammatory bowel disease
TNBS: trinitro-benzene-sulfonic acid
MPO: myeloperoxidase
IL-10: interleukin-10
TNF: tumor necrosis factor
UC: ulcerative colitis
DAI: Disease Activity Index
H&E: Hematoxylin and eosin
VGEF: vascular endothelial growth factor
MIP-α: human macrophage inflammatory protein-1α
M-CSF: macrophage colony-stimulating factor
FBS: fetal bovine serum
OUT: operational taxonomic unit
AE: alcoholic extracts
